# Stewart’s theory and acid–base changes induced by crystalloid infusion in humans: a randomized physiological trial

**DOI:** 10.1186/s13613-025-01473-9

**Published:** 2025-04-22

**Authors:** Antonio Maria Dell’Anna, Domenico Luca Grieco, Cristina Dominedò, Marta Cicetti, Irene Cisterna, Rossano Festa, Rosa Lamacchia, Giuseppina Giannì, Nicoletta Filetici, Teresa Michi, Cristina Rossi, Francesca Danila Alcaro, Alessandro Mele, Aurora Rocchi, Demetrio Del Prete, Maria Concetta Meluzio, Francesco Ciro Tamburrelli, Marco Rossi, Massimo Antonelli

**Affiliations:** 1 Department of Anesthesia and Intensive Care Medicine, Fondazione Policlinico Universitario “A. Gemelli” IRCCS, Università Cattolica del Sacro Cuore, L.Go F. Vito, 00168 Rome, Italy; 2 Department of Laboratory and Microbiological Analysis, Fondazione Policlinico Universitario “A. Gemelli” IRCCS, Università Cattolica del Sacro Cuore, Rome, Italy; 3 Department of Orthopedic and Spine Surgery, Fondazione Policlinico Universitario “A. Gemelli” IRCCS, Università Cattolica del Sacro Cuore, Rome, Italy

**Keywords:** Strong ion difference, Stewart, Chloride, Acid–base balance, Crystalloids

## Abstract

**Background:**

Stewart’s acid–base theory states that, under isocapnic conditions, crystalloid infusion affects plasma pH due to changes in strong ion difference and total weak acid concentration: a comprehensive study also assessing renal response and hemodilution effects has not been conducted in humans. We aimed to evaluate Stewart’s approach during crystalloid infusion in humans.

**Methods:**

In this randomized trial, patients undergoing surgery with minimal blood losses were randomized to receive to normal saline (chloride content 154 mEq/L, strong ion difference 0 mEq/L), lactated Ringer’s (chloride content 112 mEq/L, strong ion difference 29 mEq/L) or Crystalsol (chloride content 98 mEq/L, strong ion difference 50 mEq/L): patients received 10 ml/kg immediately after intubation, and 20 ml/kg after 2 h. Plasma/urinary acid–base and electrolytes were measured before study start and then at prespecified timepoints. The primary endpoint was pH one hour after the second fluid bolus: secondary outcomes included urinary/plasmatic electrolyte concentrations and strong ion difference during the study.

**Results:**

Forty-five patients were enrolled (15 in each group). The extent of hemodilution achieved with the first (median [Interquartile range]: saline 9% [6–15], Ringer’s 7% [4–9], Crystalsol 8% [5–12]) and the second fluid bolus (saline 13% [5–17], Ringer’s 12% [9–15], Crystalsol 15% [10–20]) was not different between groups (*p* = 0.39 and *p* = 0.19, respectively). Patients in saline group received more chloride (449 mEq [383–495]) vs. Ringer’s (358 mEq [297–419]) and Crystalsol groups (318 mEq [240–366]) (*p* = 0.001). One hour after the second bolus, pH was lower in saline group (7.34 [7.32–7.36]) vs. Ringer’s (7.40 [7.35–7.43) and Crystalsol groups (7.42 [7.38–7.44]) (both *p* < 0.01), since plasma chloride increased significantly over time in saline group but not in Ringer’s and Crystalsol groups. Overall chloride urinary excretion was not different between study groups (saline 36 mEq [28–64], Ringer’s 42 mEq [29–68], Crystalsol 44 mEq [27–56], *p* = 0.60) but, at the end of experiments, urinary chloride concentration was higher and diuresis was lower in saline group vs. Ringer’s and Crystalsol groups (*p* = 0.01, *p* = 0.04, respectively).

**Conclusions:**

Consistent with Stewart’s approach, crystalloid solutions with high chloride content lower pH due to reduced strong ion difference, progressive hemodilutional acidosis and limited renal response to chloride load.

**Trial Registration:**

Registered on clinicaltrials.gov (NCT03507062) on April, 24th 2018.

**Supplementary Information:**

The online version contains supplementary material available at 10.1186/s13613-025-01473-9.

## Background

Intravenous fluids are prescribed for different purposes in all medical and surgical settings [1, 2]. The amount of fluid administered to patients is particularly relevant in perioperative and intensive care medicine [3–5]. Crystalloids are easily available and are considered safer than synthetic colloids [6]. They are classified according to their composition to normal saline (NaCl 0.9%) and buffered solutions, which contain organic acids with negative charges such as lactate or acetate. Fluids may be used for hemodynamic optimization, rehydration and for diluting other drugs [7, 8]. However, when administered in high doses, crystalloids cause hemodilution (i.e., increase in the ratio of fluid content to both cellular and protein components of the blood) and affect plasma electrolyte composition. According to Stewart’s approach, the difference between strong cations and anions (i.e., completely dissociated), termed strong ion difference (SID), is one of the three determinants of pH, along with total plasma weak non-volatile acid concentration (Atot) and pCO_2_ [9, 10].

In vitro studies confirmed that Stewart’s physical–chemical approach is able to describe and predict the changes in pH due to dilution with crystalloids [11]. An animal study yielded the same results in terms of pH modification and showed that intact kidneys can counteract pH and electrolytes derangements induced by crystalloid intake [12]. One in vivo study explored the effect of normal saline on pH during gynecological surgery more than 20 years ago [13]; in this study, however, saline was compared to only one solution, and whether and to what extent renal response contributed to the observed results was not assessed.

We conducted a randomized trial to evaluate the effects of different crystalloids on plasmatic/urinary acid–base and electrolyte composition in healthy subjects undergoing general anesthesia, finally aiming at testing Stewart’s approach validity in humans receiving crystalloid infusion.

## Methods

### Study overview and patient selection

The “Effect of Crystalloids With Different SID on pH, and Urinary Electrolytes in humans (CRYSID)” was an investigator-initiated, single-center, open-label, three-arm randomized trial conducted in a university hospital in Italy between April 2018 and March 2021. The study protocol was approved by the Local Ethics Committee (Prot. 31979/17 (42180/17), ID 1669) and registered on clinicaltrials.gov (NCT03507062) prior to patients’ enrollment. Written informed consent to participate in the study and data analysis was obtained by all patients.

Consecutive adult American Society of Anesthesiology class I–II patients, undergoing major spine surgery with an expected anesthesia duration of at least three hours were included. Because of the long-lasting anesthesia, the minimal expected blood losses and the absence of other factors possibly interfering with determinants of acid–base state, this surgery ensured optimal experimental conditions. Main exclusion criteria were: any diuretic treatment before surgery, history of chronic kidney disease (defined as an estimated globular filtration rate < 60 ml/min/1.73 m^2^) or chronic obstructive pulmonary disease, chronic heart failure with a New York Heart Association class ≥ 2, surgery with likely fluid losses exceeding 500 ml. The full list of exclusion criteria is displayed in supplementary Table 1.

### Patient management

All enrolled patients underwent general anesthesia and were placed in prone position during the entire procedure, underwent standard monitoring including continuous five-lead electrocardiogram, pulse oximetry and invasive arterial blood pressure. Anesthetic depth was continuously monitored by the bi-spectral index, and neuromuscular blockade was assessed through train-of-four monitoring. Body temperature was monitored by a dedicated esophageal probe.

The detailed description of the standardized anesthetic and hemodynamic management is provided in the Supplementary material. Briefly, before admission to the operating room, a slow infusion of 500 mL of lactated Ringer’s was administered to account for pre-operative fasting. Infused fluids were taken into account for the purpose of the study. Anesthesia was commenced with intravenous induction and maintained with volatile agents. Analgesia was guaranteed by administration of fentanyl or sufentanyl, and muscle paralysis by rocuronium bromide. All patients were intubated and connected to mechanical ventilator in volume-control mode with tidal volume of 7 mL of predicted body weight and positive end-expiratory pressure of 5 cmH_2_O. FiO_2_ was set to achieve a peripheral oxygen saturation > 94% and respiratory rate was titrated to obtain a PaCO_2_ between 35 and 45 mmHg at the beginning of surgery and throughout the study.

### Study protocol and data collection

Enrolled patients were randomized in 1:1:1 fashion through computer-generated group assignment to one of the three study groups: normal saline (NS), lactated Ringer’s (LR) and Crystalsol (CR). These three solutions have different electrolytic composition, osmolarity and pH. All chemical characteristics of the study fluids are summarized in supplementary Table 2. The main differences among solutions are related to sodium and chloride amount and the presence of strong organic acid rapidly metabolized after infusion by the liver, restoring a new physical–chemical equilibrium, according to Stewart’s approach.

The assigned solution was the only administered throughout the procedure. If other drugs, incompatible with study crystalloid, were needed, 5% glucose was prescribed as diluting agent. Attending anesthesiologist was aware of patient allocation. Outcome assessors and statistician were unaware of study group assignment.

Study protocol is illustrated in Supplementary Fig. 1. After prone positioning and surgery initiation, a baseline (T0) blood sample was drawn for arterial blood gas analysis and chemistry including albumin, creatinine, blood urea nitrogen and phosphate, along with a urine sample for urinary electrolytes (Na^+^, K^+^, Cl^−^) and creatinine, measured in the central lab by a dedicated machine (A De Mori ABLFlex 800, Milan, Italy). Afterwards, the first fluid bolus of 10 mL/kg of assigned crystalloid solution was administered in ten minutes adopting a pressure bag. Five minutes after (T1) the end of the fluid bolus another, blood gases and chemistry were analyzed. One hour after the fluid bolus the same urine and blood samples of T0 were drawn (T2). One hour later, the same scheme was replicated (T3–T4–T5) with a fluid bolus of 20 mL/kg in 20 min. Hourly urine output was measured.

Routine maintenance fluid was administered with the assigned solution a rate of 1–2 mL/kg/h.

Demographics, surgical and anesthesiologic data were collected. At each timepoint, data regarding respiratory (tidal volume, respiratory rate, PEEP, peak pressure, plateau pressure, driving pressure, EtCO_2_), hemodynamic (heart rate, arterial pressure) data were collected. Administered fluids and urine output, acid base equilibrium and urinary electrolytic composition were recorded on an hourly basis. Based on these data, SID was calculated. According to Stewart’s theory, SID measured as the difference of strong ions (Na^+^ + K^+^ + Ca^2+^ + Mg^2+^–Cl^−^–lactate) is defined apparent SID (SIDa), while SID measured as the sum of non-volatile weak acids is defined effective SID (SIDe). SIDe is the sum of bicarbonate, phosphate and albumin, which behave as weak acid in human plasma because of their negative charges. Strong ion gap (SIG) was calculated as the difference between SIDa minus SIDe (details in the Supplementary appendix).

### Endpoints

The primary endpoint of the study was plasma pH after one hour from the second fluid bolus. Secondary endpoints included: of SIDa, SIDe, base excess (BE), strong plasma and urinary electrolytes (sodium, potassium, chloride and lactate) concentration and urinary SID (SIDu) over time.

### Statistical analysis

Continuous variables are expressed as median and interquartile range (IQR). Two independent samples were compared with Mann–Whitney test, more than two independent samples with Kruskal–Wallis test. Wilcoxon sum rank test was used to analyze two repeated variables and Friedman’s ANOVA more than two repeated variables. In order to compare the time course of the three groups (NS, LR and CR) over time, three-way ANOVA analysis and mixed model analysis imputing for missing values, were adopted; Dunn’s correction was applied for multiple comparisons.

Categorical data are displayed as number of events and percentage (%) and analyzed with Fisher’s exact test or chi square test, as appropriate.

In order to assess the hemodilutional effect of infused crystalloids, hemodilution was estimated based on changes in albumin and hemoglobin % concentrations, assessed five minutes after each bolus (Supplementary appendix). Global hemodilution was considered as the mean of the two measures. The load of Na^+^, Cl^−^ and K^+^, administered to each patient was calculated multiplying the volume of infused crystalloid at each time point expressed in liters by the concentration of the electrolyte (i.e., for 500 mL of normal saline, Na^+^ amount = 154 mEq/L * 0.5 L = 77 mEq). The total administered amount of each electrolyte was calculated. Similarly, total urinary electrolytes amount was calculated multiplying urinary concentration by urine volume in that time frame (i.e., diuresis). The sum of each single time point yielded total amount of urinary electrolyte excretion during the study period. Chloride retention was calculated as the difference between total administered chloride amount and excretion.

Fractional sodium (FENa) and chloride (FECl) excretion were calculated as the ratio of the product of urinary electrolyte concentration and serum creatinine to the product of serum electrolyte concentration and urinary creatinine [14] (Supplementary Appendix).

Estimated glomerular filtration rate (eGFR) was calculated with CKD-EPI [15] formulas, in order to estimate GFR through study procedure. All analysis were conducted blinded to group assignment, which was disclosed only after the end of the process for manuscript preparation. Results with two-sided *p* ≤ 0.05 were considered significant. Statistical analysis was performed with SPSS 28.0 (IBM Corporation, Armonk, NY, USA) and GraphPad Prism 9 (La Jolla, CA, USA).

With an α error of 0.05 and a β error of 0.8, fifteen patients per group are needed to demonstrate a pH-reduction of 0.05 between patients receiving normal saline compared to lactated Ringer’s [12] and an increase in pH of 0.05 between patients treated with crystalsol compared to those treated with 0.9% saline [12]. Hence, we planned to enroll a total of 45 patients.

## Results

### Study population

**Fig. 1:**
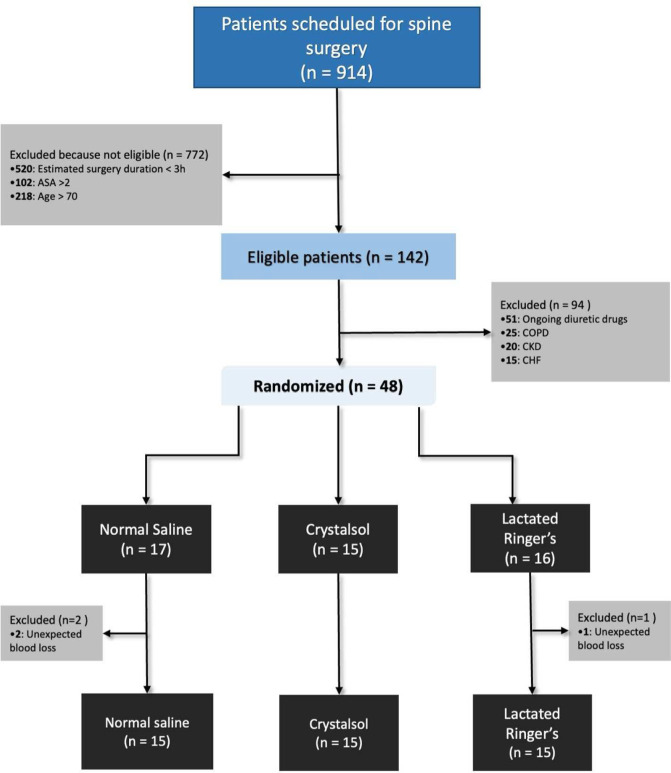
Consort diagram

Between May 2018 and June 2022, 48 consecutive patients were enrolled (Fig. 1). Enrollment was interrupted from March 2020 through March 2021 due to the surge of COVID-19.

Three patients were excluded after enrollment because of blood losses exceeding 500 ml and requiring administration of fluids and blood products not foreseen by the study protocol (two from NS and one from CR group).

Forty-five patients were included for final analysis, 15 per group; their demographics and baseline characteristics are reported in Table 1 and supplementary Table 3.

**Table 1:** General characteristic of study population

	Normal saline	Lactated Ringer’s	Crystalsol	*p* value
Age, median (IQR)	49 (42–56)	60 (44–68)	48 (32–65)	0.45
Sex, male *n* (%)	6 (40)	9 (60)	6 (40)	0.44
Weight, kg median (IQR)	70 (55–80)	71 (66–88)	80 (58–87)	0.76
Height, cm median (IQR)	166 (160–173)	170 (163–180)	170 (163–180)	0.72
BMI, kg/m^2^ median (IQR)	25 (20–28)	25 (24–27)	26 (21–28)	0.98
ASA, median (IQR)	2 (1–2)	2 (2–2)	2 (1–2)	0.49
Anesthesia duration, min median (IQR)	330 (270–400)	292 (269–349)	347 (300–540)	0.26
Surgery duration, min median (IQR)	250 (200–340)	240 (200–340)	312 (235–455)	0.25
Serum Creatinine, mg/dl median (IQR)	0.71 (0.47–0.78)	0.76 (0.6–0.86)	0.66 (0.57–0.75)	0.47
eGFR, mL/min/1.73m^2^ median (IQR)	109 (96–118)	104 (86–119)	113 (95–128)	0.80
Charlson ‘s Comorbidity Index, median (IQR)	1.0 (0.0–1.0)	1.0 (0.0–2.2)	1.0 (0.0–2.00)	0.90
Blood losses, ml median (IQR)	130 (0–300)	150 (40–320)	260 (150–320)	0.26
pH, median (IQR)	7.42 (7.39–7.43)	7.42 (7.38–7.45)	7.43 (7.4–7.46)	0.53
SIDa, mEq/L median (IQR)	33.7 (32.3–35.6)	32.9 (31–35.7)	34.4 (31.3–38.1)	0.74
Atot, mEq/L median (IQR)	14.2 (13.9–15)	13.6 (13.2–14.4)	14.3 (13–14.6)	0.38
pCO_2_, mmHg median (IQR)	33.4 (31–36.7)	34.2 (31.5–38.7)	34 (31–36.5)	0.77
BE, mmol/L median (IQR)	−2.7 (−4.5, −0.6)	−3.2 (−3.9, −0.9)	−1.6 (−3.5, 0.5)	0.44

### Plasmatic acid–base equilibrium

**Fig. 2:**
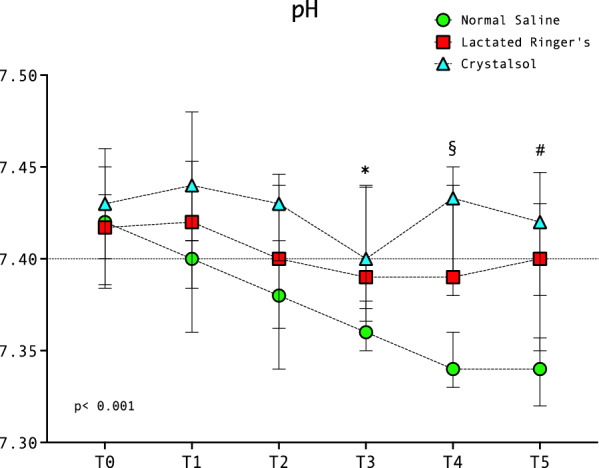
pH changes in NS, LR and CR group throughout study period. Data are presented as median and interquartile range. NS = normal saline, LR = lactated Ringer’s, CR = Crystalsol. *NS vs LR, *p* = 0.04; NS vs CR, *p* = 0.03. §NS vs LR, *p* = 0.001; NS vs CR, *p* < 0.001. # NS vs LR, *p* = 0.008; NS vs CR, *p* < 0.001

Plasma pH at the last timepoint of the study (T5) was significantly lower in NS (7.34 [7.32–7.36]) compared to LR (7.40 [7.35–7.43], *p* = 0.008) and CR (7.42 [7.38–7.44], *p* < 0.001). Compared to LR and CR groups, plasma pH in NS group was significantly lower at T3 and T4 as well (Fig. 2).

**Fig. 3:**
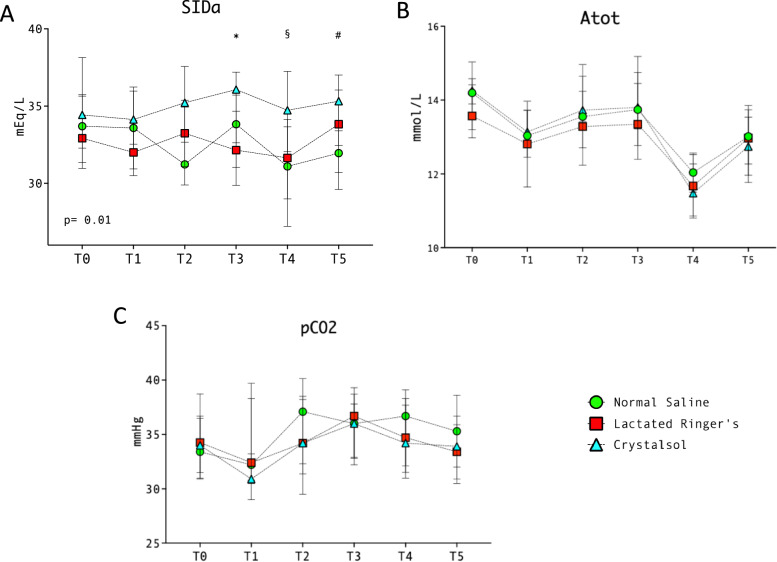
Changes in SIDa, Atot and pCO_2_ among the three groups. Green circles: normal saline (NS); red squares: lactated Ringer’s (LR); sky blue triangles: crystalsol (CR). **A** *NS vs CR, *p* = 0.018; LR vs CR, *p* = 0.024. §NS vs CR, *p* = 0.003; LR vs CR, *p* < 0.001. # NS vs LR, *p* = 0.04; NS vs CR, *p* = 0.002. **B.** All comparisons had *p* > 0.05. **C** *NS vs CR, *p* = 0.04; LR vs CR, *p* = 0.024. §NS vs CR, *p* < 0.001; LR vs CR, *p* = 0.02. # NS vs LR, *p* = 0.02; NS vs CR, *p* = 0.003

SIDa and SIDe were significantly reduced at T5 in patients receiving NS (32 [29–33] and 29 [27–31]) compared to CR (35 [32–37] and 32 [30–34], *p* = 0.002 and *p* = 0.003, respectively, Fig. 3 and Supplementary Fig. 2). Strong ion gap did not differ significantly across the time points (Table 2), while base excess decreased significantly in NS group (T0 −2.7 [−4.5, −0.6]; T5 −5.9 [−7.7, −4.9], *p* < 0.001) and was significantly lower at T5 compared to LR (−2.7 [−4.5, −0.6], *p* = 0.02) and CR (−3 [−4, −1.6], *p* = 0.003). PaCO_2_ and total concentration of non-volatile acids (Atot) did not show significant changes throughout study period (Fig. 3).

**Table 2:** Electrolytes, SIG and BE variation over time

Variable		T0	T1	T2	T3	T4	T5	*p* value
Na^+^	Saline	140 (138–143)	140 (139–144)	141 (139–143)	140 (139–144)	140 (140–143)	140 (138–143)	0.18
	LR	141 (138–142)	140 (138–142)	141 (139–143)	140 (138–143)	139 (138–141)	140 (138–144)	
	CR	141 (140–142)	141 (138–143)	141 (140–143)	141 (139–144)	140 (138–144)	141 (138–143)	
Cl^−^	Saline^**^	109 (107–110)	111 (109–113)	111 (110–112)^*^	110 (109–113)^*^	113 (111–115)^*^	111 (109–115)^*^	** < 0.001**
	LR	109 (107–110)	110 (106–111)	110 (108–111)	110 (108–112)	110 (107–112)^§^	109 (107–111)	
	CR	109 (106–110)	108 (107–111)	108 (106–110)^#^	108 (107–111)^#^	108 (106–110)^#^	108 (107–110)^#^	
K^+^	Saline^**^	3.6 (3.5–3.9)	3.5 (3.2–3.7)	3.7 (3.4–4.0)	3.9 (3.4–4.3)	3.7 (3.5–4.1)	4.1 (3.7–4.5)	0.14
	LR^**^	3.7 (3.5–3.8)	3.7 (3.6–4.0)	3.8 (3.7–4.1)	4.0 (3.8–4.2)	4.0 (3.8–4.1)	4.1 (3.9–4.6)	
	CR^**^	3.8 (3.2–4.0)	3.6 (3.2–4.0)	3.8 (3.4–4.2)	3.9 (3.6–4.4)	3.8 (3.6–4.2)	3.8 (3.6–4.3)	
SIG	Saline	1.7 (−1.6,3.2)	2.3 (−1.4,3.2)	1.0 (−2.1,3.0)	1.2 (−2.7,4.9)	2.5 (−0.8,5.0)	2.6 (−0.9,5.2)	0.47
	LR	0.8 (−1.2,2.4)	1.0 (−1.4,3.2)	1.3 (−1.1,3.7)	0.5 (−1.6,1.6)	0.5 (−1.7,2.9)	2.3 (−0.7,5.5)	
	CR	1.1 (−0.6,4.3)	2.8 (−0.6,3.5)	2.4 (0.3,3.8)	1.6 (−1.2,4.6)	2.4 (0.9,6.0)	1.0 (−0.3,6.5)	
BE	Saline^**^	−2.7 (−4.5,−0.6)	−3.6 (−5.9,−2.6)	−3.6 (−7.1,−2.4)	−3.8 (−6.8,−2.6)	−5.7(−7.7,−4.6)^*^	−5.9 (−7.7,−4.9)^*^	0.09
	LR	−3.2 (−3.9,−0.9)	−2.8 (−4.0,−1.0)	−2.8 (−4.6,−1.3)	−3.6 (−4.9,−1.3)	−3.7 (−4.9,−2.3)^§^	−2.7 (−4.5,−0.6)^§^	
	CR^**^	−1.6 (−3.5,0.5)	−2.0 (−4.1,−1.0)	−2.6 (−4.5,−1.9)	−2.8 (−4.0,−1.7)	−2.1 (−4.2, −0.3)^#^	−3.1 (−4.1,−1.6)^#^	

All data are represented as median and interquartile range. Sodium, potassium and chloride and SIG are expressed as mEq/L, BE as mmol/L. *p* value reports the overall significance of mixed linear model adopted

*LR* Lactated Ringer’s, *Cr* Crystalsol, *SIG* strong ion gap, *BE* base excess

^*^p < 0.05 for overall comparison among the three groups

^**^p < 0.05 for change within the same group

^#^*p* < 0.05 for comparison between NS and Cr

^§^*p* < 0.05 for comparison between NS and LR

^$^*p* < 0.05 for comparison between LR and Cr

### Electrolytes and fluids administration

**Fig. 4:**
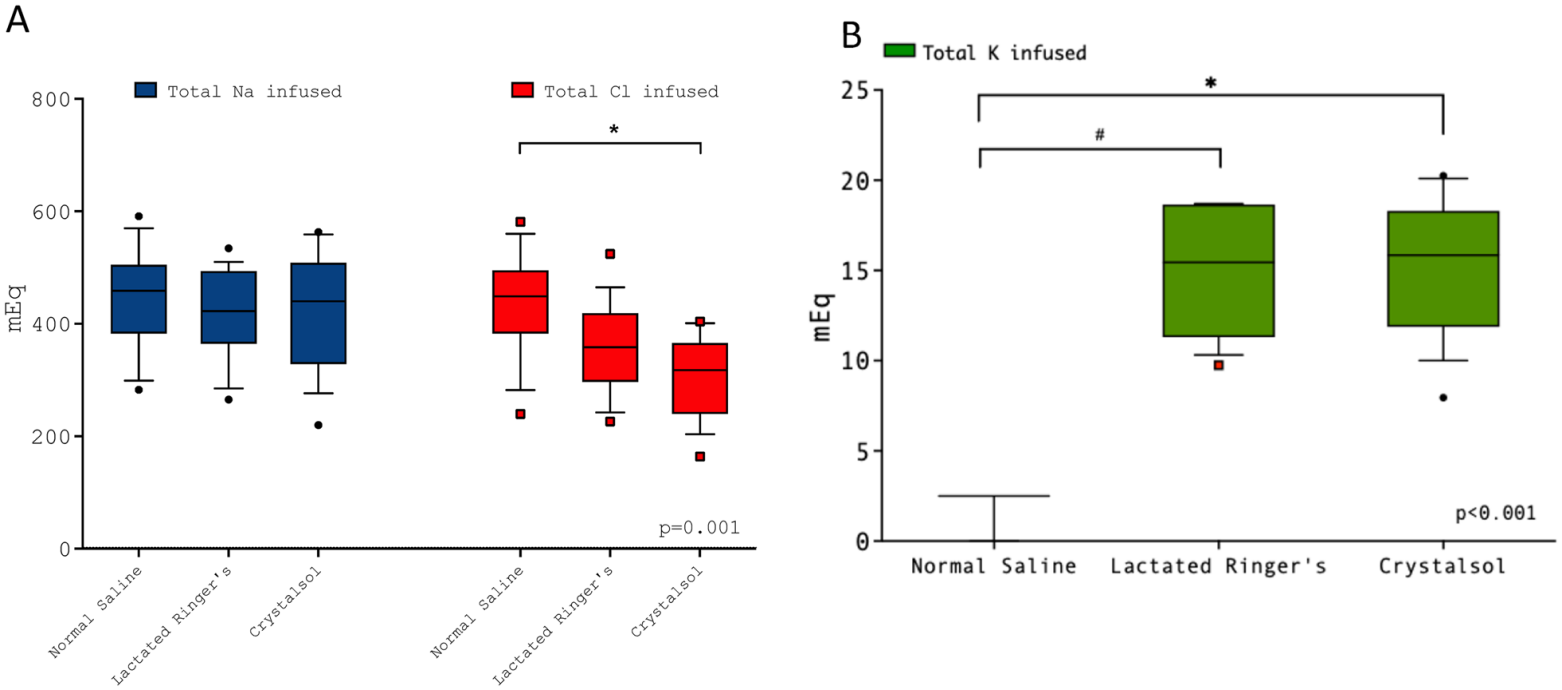
Total amount of infused sodium, chloride and potassium. Sodium infused: NS 459 (383–505), LR 422 (365–494), CR 440 (328–508) mEq. Chloride infused: NS 449 (383–495), LR 360 (297–419), CR 318 (240–366) mEq. Potassium infused: NS 2.5 (2.5–2.5), LR 15.5 (11–19), CR 16 (12–18) mEq; *p* < 0.001. *p < 0.001; **#***p* < 0.001

The total amount of administered crystalloids was not different between groups (Supplementary Fig. 3–5), also when it was calculated hourly (saline 16 [15–17] vs LR 14 [14–17] vs CR 15 [15, 16] mL/kg/h, *p* = 0.28). Median total administered sodium during the study period was comparable among study groups (NS 459 [383–505] mEq, LR 422 [365–494] mEq, CR 440 [328–508] mEq, *p* = 0.79, Fig. 4), while total administered chloride was significantly higher in normal saline group (NS 449 [383–495] mEq, LR 358 [297–419] mEq, CR 318 [240–366] mEq, *p* = 0.001, Fig. 4). Administered potassium was significantly lower in NS group (NS 2.5 [2.5–2.5] mEq, LR 15.4 [11.3–18.7] mEq, CR 15.8 [11.9–18.3] mEq, *p* < 0.001, Fig. 4).

Hemodilution was achieved in all three groups without significant differences among groups both after the first (NS 9.4 [6–14.5], LR 6.7 [4–8.8], CR 7.9 [4.9–11.5]%, *p* = 0.39, Supplementary Fig. 6 and 16) and the second bolus (NS 12.9 [5.4–16.8], LR 11.5 [9.2–15], CR 14.8 [10–20]%, *p* = 0.19, Supplementary Fig. 6 and 16). Global hemodilution was significantly higher after the second compared to the first bolus (first bolus 7.9 [5.4–11.4], second bolus 12.8 [8.7–17.7] %, *p* < 0.001, Supplementary Fig. 6). Plasmatic concentration of sodium was substantially unchanged across the time points among the three groups (Table 2). Plasma chloride increased significantly in NS group from T0 to T5 (109 [107–110] and 111 [109–115], *p* < 0.001, Table 2). Potassium concentration significantly increased in all three groups, from T0 to T5, but without significant inter-group differences (NS *p* < 0.001, LR *p* < 0.001, CR *p* = 0.005, Table 2).

### Urinary electrolytes and renal function

Urinary sodium and potassium concentrations were not different between study groups at any timepoint (Fig. 5). Urinary chloride was significantly higher in NS compared to CR at T5 (86 [46–146] mEq/L vs 30 [18–85] mEq/L, respectively, *p* = 0.01, Fig. 6B). Urinary SID was significantly higher in CR group compared both to NS and LR at T5 (102 [55–112] vs 23 [11–33] vs 14 [4–51] mEq/L, respectively, *p* = 0.006, *p* < 0.001, Fig. 5 and Supplementary Figs. 7–16).

**Fig. 5:**
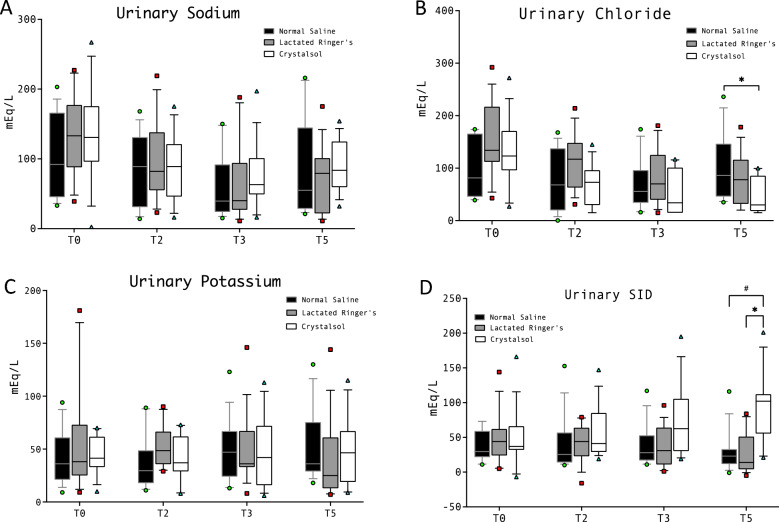
Urinary concentration of sodium, chloride and potassium and SID. **A **All inter-group differences were non significant. **B** *NS 86 (46–146) CR 30 (18–85) mEq/L, *p* = 0.01. **C **All inter-group differences were non significant. **D** CR 102 (55–112) vs NS 23 (11–33) vs LR 14 (4–51) mEq/L; **#***p* = 0.006; *p < 0.001

**Fig. 6:**
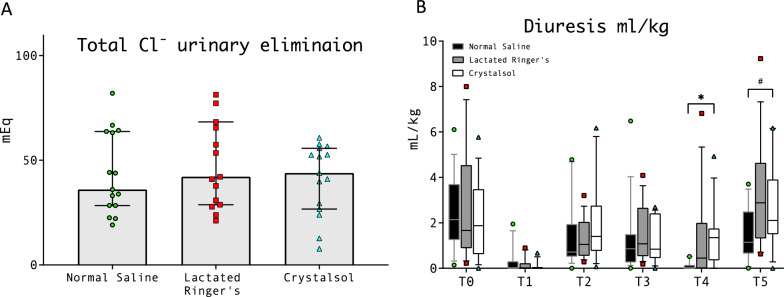
Total chloride elimination among groups and diuresis across time point. **A** Total Cl urinary elimination NS 36 (28.4–63.7), LR 42.1 (28.8–68.3), CR 43.9 (26.7–55.7) mEq. **B** *NS 0 (0–0.14) LR 0.4 (0–2) CR 1.3 (0.3–1.7) *p* = 0.002 (*p* = 0.001); # NS 1.1 (0.6–2.5) LR 2.9 (1.3–4.6) CR 2.1 (1.5–3.9) *p* = 0.04

Serum creatine was comparable among groups across all the time points (Supplementary Fig. 10). Total chloride urinary elimination was similar between groups (NS 36 [28–64], LR 42 [29–68], CR 44 [27–56] mEq, *p* = 0.60, Fig. 6). Diuresis was significantly lower in NS group both at T4 (NS 0 (0–0.14) LR 0.4 (0–2) CR 1.3 (0.3–1.7), mL/kg *p* = 0.002) and T5 (NS 1.1 (0.6–2.5) LR 2.9 (1.3–4.6) CR 2.1 (1.5–3.9) mL/kg, *p* = 0.04, Fig. 6).

## Discussion

In this randomized trial, we demonstrate that the pH reduction produced by normal saline compared to lactated ringer’s and crystalsol is mechanistically caused by a reduction in SIDa combined with hemodilution [16, 17]. This is the first human study to test the validity of Stewart’s approach to acid–base balance, including the analysis of renal response to different crystalloids and hemodilution degrees.

The mechanistic role of chloride excess in reducing plasmatic SIDa was proposed by Gattinoni et al. in a seminal work [18]. The concept of “dilutional acidosis” was then introduced; this refers to acidosis due to dilution of both SIDa and Atot after administration of any solution with SID 0 (i.e., pure water or 0.9% saline) in isocapnic conditions. In our population, we assessed the role of hemodilution after infusion of two different fluid amounts. The decrease in pH was more pronounced after the second (larger) bolus [19]. In addition, patients in NS group showed progressive SIDa and Atot reduction after each fluid bolus. Our study demonstrates in humans the concept of dilutional acidosis, previously theorized in bench experiments [18] and in an animal study [12, 20]. In these previous experiments, the authors infused a higher volume of fluids compared to our study (10% of animal body weight vs 3–5% of body weight). Nonetheless, the reported effect was comparable. This expands the relevance of these results to the clinical setting.

SIDa reduction is necessarily due to either an increase in chloride without sodium changes, or sodium reduction without chloride changes. In our population, we do not report significant changes in plasmatic sodium concentration, because the amount of infused sodium was similar in the three groups. Hence, SIDa reduction was attributable to chloride increases, as often observed in clinical practice. Moreover, a certain degree of chloride accumulation contributed to such effect. Indeed, patients assigned to NS, received more chloride without a parallel increase in urinary chloride elimination. Even if patients in NS group had a slight increase of urinary chloride at the end of the study, fraction of excreted chloride and total chloride elimination did not differ among groups. After calculation of total chloride retention as the difference between total administered and excreted chloride, we found a significant excess in chloride retention in NS group. This indicates that infused chloride overcame renal compensatory capabilities. The role of excessive chloride load on nephron has been widely studied [19]. Chloride load yields reduction of glomerular filtration rate, which impairs chloride urinary elimination, finally reducing plasmatic SIDa. A previous study [21] demonstrated that 0.9% saline can reduce renal blood flow in healthy volunteers, compared to Crystalsol. We did not study renal blood flow because of the type of surgery, and the prone position hampered doppler execution. However, patients receiving NS showed some initial degree of renal impairment, as suggested by reduced urinary output at the end of the study. Urinary chloride did not change over time in patients in NS group and a slight but significant SIDu increase was recorded one hour after the second bolus in patients receiving CR, driven by urinary chloride decrease. Such physiological observations, in a healthy population, would corroborate the data of randomized trials, that showed an increase in the incidence of acute kidney injury in critically ill patients receiving NS [22, 23]. The role of chloride in tubule-glomerular feedback and in reducing renal blood flow is well established [24–26]. We provide new evidence that such mechanisms are likely to affect healthy humans undergoing general anesthesia and receiving a relative low amount of fluids. Yet, in spite of a robust physiological rationale, other randomized trials [27–29] did not corroborate the physiological evidence of a detrimental effect of NS on clinical outcome in critically ill patients [30, 31]. Our findings suggest that a relevant volume of crystalloid over a short period of time (40–50 ml/kg in a time frame of 4 h) may induce small but significant changes in pH and urinary response to chloride load. Integrating these results to those of large clinical trials would indicate that lower infused volumes over a longer period (i.e., 2.5 L over 48 h) may instead not be able to affect patient’s physiology and clinical outcomes.

The small but significant change in pH was likely responsible for the observed increase in plasmatic potassium in NS group, as previously observed in the context of kidney transplantation [32, 33]. Indeed, normal saline does not contain K^+^, and potassium urinary elimination was comparable among groups.

The change in pH according to the volume and type of infused solution roughly confirmed the experimental results of Carlesso et al. [11]. Yet, our study highlights one potential limit of Stewart’s theory application in vivo; the alkalinizing effect of CR was indeed less evident in our population, compared to their experimental findings, while the acidifying role of NS was confirmed. We could speculate that surgery may itself induce some sort of acidosis due to local tissue dysoxia and/or surgical stress [34], which happens to be counterbalanced by solutions with higher SID and exacerbated by normal saline. Also, the renal system seems more able to counteract alkalinization than acidification, as shown by the significant increase in SIDu after administration of CR.

Our study has limitations. First, a small number of patients were enrolled in a well pre-specified scenario. Because of this limited sample, no adjustment for multiple comparisons nor multivariable analysis were performed. However, the limited sample allowed thorough control of all study variables, which is essential for a physiologic study. Second, patients were monitored for a short period of time (i.e., 3–5 h) and we cannot infer the possible impact of infused crystalloid on longer-term outcomes. We speculate that long-term effects of infused crystalloids would have been unlikely, and the focus of our work was to ascertain immediate physiological effects of different crystalloid solutions. Three different crystalloids were chosen, normal saline and two buffered solutions with different composition and SID. We cannot extend our results to other types of commercially available fluids (i.e., acetated Ringer’s), even if the impact of small differences in the buffer acid should be negligible in healthy people [35, 36]. Also, we enrolled patients undergoing surgery ; hence, the effect of surgery and anesthesia on kidney function should have been taken into account as a possible confounder [37]. Nonetheless, our findings seem consistent with those of previous physiological studies conducted on awake people. Finally, we could not measure directly glomerular filtration rate. Because of the short time frame of our study, creatinine-based tools cannot detect renal injury, as shown in time course of creatinine levels and estimated glomerular filtration rate. Also the decrease of urinary output should be interpreted with caution, because the present paper was not designed for this purpose and confounders as well as interindividual variability may account for the observed results.

## Conclusion

Intravenous infusion of crystalloid solutions with high chloride load reduces pH by hemodilution and changes in plasma SID caused by urinary chloride retention. Normal saline induced significant reduction in diuresis, with excess in plasma chloride not fully counteracted by the kidney. This randomized trial confirms that Stewart’s approach rules acid–base effects of crystalloid infusions in humans.

## Supplementary Information

Additional file 1

## Data Availability

The datasets used and/or analyzed during the current study are available from the corresponding author on reasonable request.

## Abbreviations

SID: Strong ion difference
SIDa: Apparent stron ion difference
SIDe: Effective strong ion difference
SIDu: Urinary strong ion difference
FENa: Fractional sodium excretion
FECl: Fractional chloride excretion
Atot: Plasma weak acid concentration
Na: Sodium
K: Potassium
Cl: Chloride
NS: Normal saline
CR: Crystalsol
LR: Lactated Ringer’s
eGFR: Estimated glomerular filtration rate
