# Supplementary material for: Stewart’s theory and acid–base changes induced by crystalloid infusion in humans: a randomized physiological trial
Source: Ann Intensive Care. 2025 Apr 22;15:54. doi: 10.1186/s13613-025-01473-9 (PMC12014888; doi:10.1186/s13613-025-01473-9)
Supplement: Supplementary file 1 — Additional file 1 [file 13613_2025_1473_MOESM1_ESM.docx]

**Supplementary Appendix**

**Protocol for general anesthesia**

All patients received standard monitoring including continuous ECG, SpO_2_ and continuous arterial pressure recorded via radial arterial line. Uncalibrated arterial pulse-contour (Vigileo - Edwards Lifesciences, Irvine, Ca) was considered by treating clinician, for advanced hemodynamic monitoring if appropriate. Depth of anesthesia was provided continuously by the bispectral index (BIS) whereas neuromuscular blockade was assessed via the TOF (train of four) monitoring. Esophageal temperature was monitored, as well.

Two venous access were placed, at least; one for fluid and one for drug administration. Fluid infusion was performed through a 18 G cannula in all patients.

General anesthesia was induced in line with current practice with Propofol 1.5-2.5 mg/kg iv, Midazolam 0.2-0.4 mg/kg iv or Ketamine 1-2 mg/kg iv, as hypnotic agents; Fentanyl 1-2 mcg/kg iv or sufentanyl 0.1-0.3 mcg/kg iv, as opioids; Rocuronium 0.4-0.6 mg/kg iv or cisatracurium 0.15-0.20 mg/kg, as neuromuscular blocking agents.

Anesthesia maintenance was performed using an inhaled agent, sevoflurane (MAC 0.8-1.2 or 1.4-2.6%) or desflurane (MAC 0.8-1.2 or 5-7.25%). Intraoperative analgesia was guaranteed by fentanyl 1-2 mcg/kg iv prn, sufentanyl 10-50 mcg iv prn, or continuous infusion of remifentanil 0.05-0.30 mcg/kg/min. Paralysis was maintained with rocuronium 0.1-0.2 mg/kg iv prn or cisatracurium 0.03 mg/kg iv prn or a continuous infusion of 1-2 mcg/kg/min.

Patients were ventilated in volume control mode (VC) with a tidal volume (TV) of 6-8 mL/kg (predicted body weight, using the following formula: 50 + 0.91 (height [cm] −152.4) for men and 45.5 + 0.91 (height [cm] −152.4) for women)[1] and a positive end-expiratory pressure (PEEP) of 5 mmHg. FiO_2_ was set to achieve a peripheral oxygen saturation (SpO2) > 95%. At baseline, PaCO_2_ was compared to end-tidal carbon dioxide (EtCO_2_) and the last one was regulated according to PaCO_2_ value. During all the surgery, respiratory frequency and minute volume ventilation were set to maintain EtCO_2_ in a normal range (35-45 mmHg).

**Strong ion difference meaning and Stewart approach to acid-base equilibrium**

Strong ion difference (SID) has been defined as the difference between strong ions with a positive and a negative charge [2, 3]. A strong ion is a molecule which is entirely dissociated in aqueous solution. A clear example is the common salt (sodium chloride, NaCl), which is completely dissociated in Na^+^ and Cl^-^, when dissolved in water. When only strong ions are present in a solution, SID is always 0 (i.e. normal saline 0.9%: Na^+^ 154 mEq, Cl^-^ 154 mEq, SID =0).

Human plasma is a more complex solution, where strong ions, weak non-volatile acid (Atot) and volatile acid (carbon dioxide, CO_2_) coexist. In healthy condition normal plasmatic SID is approximately 40-42 mEq[2, 3]. Stewart showed in the 70’s that in experimental conditions pH of an aqueous solution (i.e. H^+^ concentration) can be manipulated by changing weak non-volatile acid concentration (Atot= AH + A^-^), partial pressure of CO_2_ (pCO_2_), and SID. More specifically, Stewart demonstrated that classical Henderson-Hasselbach approach at acid-base equilibrium (ABE), is more a descriptive approach than a causal approach and its validity is confirmed only in situation in which SID and AH remain constant. From this perspective, [OH-] and [HCO_3_^-^] become dependent variables, related to the three independent variables, regulating ABE.

Plasmatic SID can be calculated by simply subtracting strong anions (Cl^-^ and lactate) to strong cations (Na^+^, K^+^, Ca^2+^, Mg^2+^), yielding the so-called apparent SID (SIDa). Besides SIDa, also effective SID (SIDe) can be calculated, taking into account all the weak non-volatile acid with a negative charge, necessarily present in plasma in order to guarantee electroneutrality (i.e. the same total amount of positive and negative charges). Both the solutions available for iv infusion and organic fluids have necessarily the same amount of positive and negative charges. However, when weak non-volatile acids are present, SID becomes positive. For commercially available fluids, SID corresponds exactly to the amount of organic acids, because they are completely metabolized after infusion, mostly in bicarbonate. So, for example, Lactated Ringer’s has a SID of 29 mmol/L and Plasmalyte, Hartmann solution and Crystalsol of 50 mmol/L.

Conceptually the most relevant non-volatile weak acids in plasma are albumin and phosphate (PO_4_^3-^), even if they would include hemoglobin (Hb), which exerts a more pronounced role in whole blood and sulfate (SO^3-^), as well. The formula (3) allows to measure the Atot, accounting for negative charge contribution of albumin (expressed in g/L) and phosphate, in addition to bicarbonate (HCO^3-^), which are calculated based on pCO_2_ and pH as follows (1) [4]:

1. [HCO^3-^]= 0.0306*pCO_2_*10^(pH-p^*^K^*^a)^[5]
2. [PO_4_^3-^] mmol/L= [PO_4_^3-^] mg/dL * 0.323
3. SIDe = [HCO^3-^] + [PO_4_^3-^]*(0.309*pH-0.469) + [Albumin]*(0.1204*pH-0.625)= [HCO^3-^] + [A^-^]

PCO_2_ is expressed in mmHg, [PO_4_^3-^] in mmol/L and [Albumin] in g/L. [PO_4_^3-^] was converted from mg/dL to mmol/L according to the formula (2).

The difference between SIDa and SIDe has been defined strong ion gap (SIG), representing the amount of unmeasured anions when is positive, or cations, when is negative [ref].

SIDa can be measured also in urinary sample (SIDu) by subtracting [Cl^-^] to [Na^+^] and [K^+^][6].

Atot were calculated according to the following formula[4]:

1. Atot (mmol/L)= [A-]*[1+10^(pKa-pH)^]

Where p*K*a=-log_10_1.05*10^-7^.

**Hemodilution, Electrolytes amount and urinary excretion, eGFR**

Hemodilution (HD) was calculated as the mean of percentage variation of albumin (HD_alb_) and hemoglobin (HD_Hb_) concentration 5 minutes after fluid bolus, equation (5)

1. HD = (HD_alb_ + HD_Hb_)/2
2. HD_alb_= ([albumin]_baseline_ – [albumin]_5 min after bolus_)*100/2
3. HD_Hb_= ([Hb]_baseline_ – [Hb]_5 min after bolus_)*100/2

The aforementioned calculation was repeated both for the first and the second fluid bolus. Global hemodilution was considered the mean between the two measures.

Fractional excretion of sodium (FENa)

1. FENa= $\frac{\left[ {Na}^{+} \right]_{urine}* \left[ creatine \right]_{plasma}}{\left[ {Na}^{+} \right]_{plasma}* \left[ creatine \right]_{urine}}$ *100

Fractional excretion of chloride (FECl)

1. FECl= $\frac{\left[ {Cl}^{-} \right]_{urine}* \left[ creatine \right]_{plasma}}{\left[ {Cl}^{-} \right]_{plasma}* \left[ creatine \right]_{urine}}$ *100

*CDK-EPI formula*

1. eGFR= 142 * (sCr/A)^B^ * 0.9938^Age^ * 1.012 (if female)

where A= 0.9 if male and 0.7 if female; B if female and sCr≤0.7=-0,241, if sCr>0.7= -1.2; if male and sCr ≤0.9=-0.302, if sCr>0.9=-1.2[7].

Age is expressed in years and sCr in mg/dL.

*Cl retention*

Chloride retention was calculated as the difference between total chloride amount delivered to patients and total chloride eliminated by urine.

*Predicted variation of plasma Cl^-^ (DpCl^-^)*

Predicted variation of plasmatic chloride was calculated considering that Cl^-^ is largely restricted to extracellular volume (40% of total body water – TBW). Only 25% of infused chloride, enters intravascular space [8]. The following formula was adopted for calculation:

1. DpCl^-^=$\frac{\left( initial plasma \left[ \mathrm{Cl}^{-} \right]*0.08*TBW \right)+(0.25*infusate \mathrm{Cl}^{-} content)}{0.08*TBW+0.25*infusate volume}$

Where TBW is calculated as 50% of body weight in women and 60% in men. 0.08*TBW represents the plasma volume.

| Inclusion criteria | Exclusion criteria |
| --- | --- |
| Adult patients (between 18 and 70 years old) | Pregnancy |
| ASA 1 or 2 | Chronic obstructive pulmonary disease (COPD) |
| Spinal surgery under general anesthesia, intubated and mechanically ventilated; | Chronic heart failure (CHF) with a NYHA class ≥2 |
| Normal preoperative albumin | Diuretics in the pre-operative period |
| Surgery with an estimated duration of at least 3 hours | Chronic kidney disease (CKD), defined as a GFR < 60 ml/min/1.73 m2 |
|  | Diabetes mellitus treated with insulin |
|  | Myopathies |
|  | Surgery with likely fluid losses or with unexpected bleeding during surgery |

**Table S1 Inclusion and exclusion criteria**

| Crystalloids | Na^+^ | K^+^ | Mg^2+^ | Ca^2+^ | Cl^-^ | Lactate | Acetate | Gluconate | | mOsm/L | pH |
| --- | --- | --- | --- | --- | --- | --- | --- | --- | --- | --- | --- |
| **Normal Saline**  **(0.9% NaCl)** | 154 | - | - | - | 154 | - | - | | - | 308 | 4.5 - 7 |
| **Lactated Ringer’s** | 132 | 5 | - | 4 | 112 | 29 | - | | - | 282 | 5.5 - 7 |
| **Crystalsol** | 140 | 5 | 3 | - | 98 | - | 27 | | 23 | 296 | 6.5 - 8 |

**Table S2 Electrolytic composition of infused crystalloids and chemical characteristics**

| Study population general characteristics | |
| --- | --- |
| Age, median (IQR) | 50 (40-65) |
| Sex, male n (%) | 21 (46.7) |
| Weight, kg median (IQR) | 70 (61-85) |
| Height, cm median (IQR) | 170 (163-168) |
| BMI, kg/cm^2^ median (IQR) | 25 (22-27) |
| ASA, median (IQR) | 2 (1-2) |
| Anesthesia duration, min median (IQR) | 320 (270-400) |
| Surgery duration, min median (IQR) | 250 (205-342) |
| Serum Creatinine, mg/dl median (IQR) | 0.77 (0.69-0.91) |
| eGFR, mL/min/1.73m^2^ median (IQR) | 109 (98-118) |
| Charlson Comorbidity Index, median (IQR) | 1.0 (0.0-2.00) |

**Table S3 General population characteristics**

**
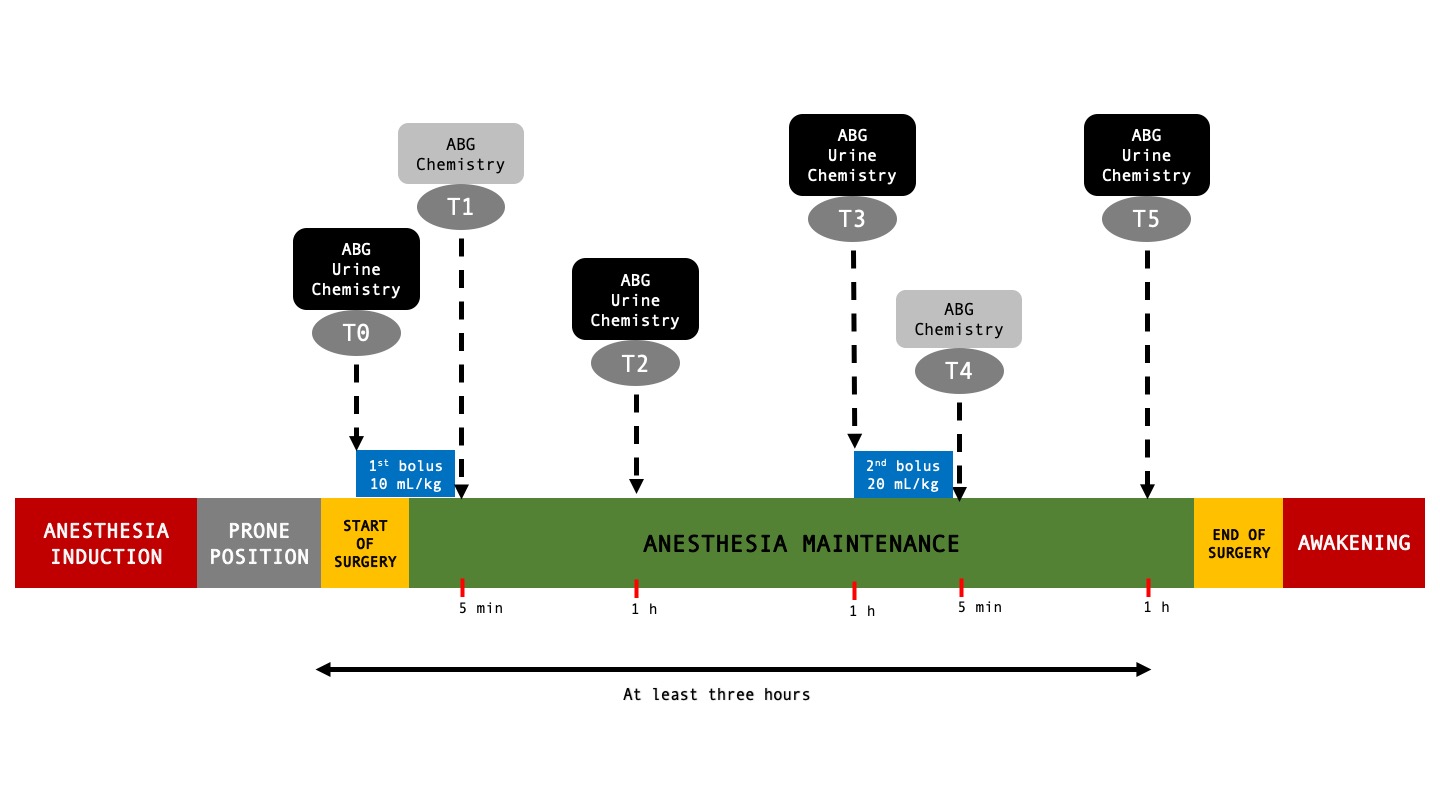
**

**Figure S1. Study protocol**

After anesthesia induction, patient was prepared for surgical procedure in prone position, then surgery began and (T0 – first ), both baseline blood sample was drawn for arterial blood gas analysis (ABG) and dosing of plasmatic albumin, creatinine, blood urea nitrogen (BUN) and phosphorus, and urine sample for urinary electrolytes (Na^+^, K^+^, Cl^-^ and creatinine). The first fluid bolus of 10 mL/kg of assigned crystalloid was infused in 10 minutes, immediately after blood samples were drawn. After five minutes from the end of infusion (T1), another blood sample was drawn for ABG and chemistry. One hour after the end of infusion (T2), ABG, plasmatic chemistry and urine were drawn. After one hour, immediately before the second fluid bolus of 20 mL/kg in 20 minutes, ABG, plasmatic chemistry and urine were drawn again (T3 – second baseline). 5 minutes after the end of the second bolus ABG and plasmatic chemistry were analyzed (T4). Finally, one hour after the end of second bolus, the last blood and urine samples were drawn for ABG, plasmatic chemistry and urine electrolytes and creatinine (T5).

A

| A   | ****  B |
| --- | --- |

**Figure S2 HCO3^-^ and SIDe**

**A Bicarbonate in plasma over time** T4 NS 20 (18-21), LR 22 (20-24), Cr 22 (21-24)# p=0.011*p<0.001; T5 NS 20 (18-20), LR 23 (20-24), Cr 22 (21-23) ##p=0.001** p<0.001

**B SIDe in plasma over time** T4 NS 29 (27-30), LR 30 (29-32), Cr 31 (30-34)NS vs Cr *p=0.002; T5 NS 30 (27-31), LR 31 (30-34), Cr 32 (30-34)§NS vs LR p=0.04, NS vs Cr p=0.002

**Figure S3 Total SID “infused”**

NS 10 (10-10) LR 64 (45-75) CR 122 (102-143) mEq, overall p<0.001; NS vs LR p=0.0046 LR vs CR 0.0046. NS vs CR p<0.001

**Figure S4 plasmatic lactate concentration throughout time points**

Dotted line at 2 mmol/L indicates the cut-off of lactate above which they are considered relevant as a marker of peripheral tissue hypoperfusion. Data are expressed in mmol/L

* NS 1 (0.8-1.6) vs RL 2.1 (1.4-2.4) p=0.008; # CR 1.2 (0.7-1.6) vs RL 2.1 (1.4-2.4), p=0.007

|   C  B |  |
| --- | --- |
|  | **Figure S5 Total fluid intake, fluid output and fluid balance**  A Total fluid intake in ml/kg across all the time points  B Total fluid output in ml/kg  C Total fluid balance in mL/kg at T5 |

A

**Figure S6 Hemodilution among study groups and time points**

A

B

**A** Hemodilution after first bolus NS 9.4 (6.1-14.5), LR 6.7 (4-8.8), CR 7.9 (4.9-11.5)%

Hemodilution after second bolus NS 12.9 (5.5-16.8), LR 11.5 (9.3-15), CR 14.8 (10-20)%

**B** Hemodilution between boluses First bolus 7.9 (5.4-11.5) second bolus 12.8 (8.7-17.7)%, p<0.001

NS=normal saline; LR= lactated Ringer’s; CR=crystalsol

|   C  A |   B |
| --- | --- |
|  | **Figure S7 Chloride infused and eliminated**  A Chloride infused calculated as mEq/mL*mL infused of study solution  B Chloride eliminated by urine calculated as mEq/L*L of urine  C Total chloride retention as the difference between total Cl^-^ infused and excreted by urine  NS 427 (377-462), LR 293 (197-365), CR 270 (229-332) p<0.001  ** NS vs RL, p=0.002; *** NS vs CR, p<0.001. |

|  |   B |
| --- | --- |

A

**Figure S8 Total SID eliminated by urine and Delta between total SID infused and excreted**

A SID excreted was calculated as the difference between total urinary cations and anions throughout all the study period.

B Delta SID was calculated as the difference between total SID infused (Fig. S3) and excreted.

** NS -0.4 (-11 - 22) vs RL 57 (37-63) p=0.009; *** NS -0.4 (-11 - 22) vs CR 102 (62-124), p<0.001

**Figure S9 Total potassium (K^+^)eliminated by urine from T0 to T5**

NS 16.7 (10.3 -18.9), RL 16 (12-25), CR 17 (14-25), p=0.86

|   A |   B |
| --- | --- |

**Figure S10 Serum creatinine and eGFR across time points**

B eGFR was calculated according to CKD-EPI formula

NS 16.7 (10.3 -18.9), RL 16 (12-25), CR 17 (14-25), p=0.86

|   A |   B |
| --- | --- |

**Figure S11 Fraction excreted of sodium and chloride**

**Figure S12 pH variation according to dilution degree and SID infused**

Data are represented as median and interquartile range

Black dotted line represents pH before the first dilution (7.42)

Yellow dotted line represents pH before the second dilution (7.39)

pH SID 0 at 8% 7.4 (7.36-7-41) and 13% 7.34 (7.33-7.36)

**Figure S13 plasma chloride actual and predicted in normal saline (NS) group**

Data are represented as median and interquartile range

Black circles represent actual plasma chloride concentration

Gray squares represent predicted plasma chloride concentration

|   A |   B |
| --- | --- |
|   C | **Figure S14 Global effect of hemodilution on SIDa, pCO_2_ and Atot**  A SIDa variation from baseline throughout hemodilution (T0-T2-T4),  Baseline 33.7 (32-36), 8% dilution 33.3 (31.7-35.6), 13% dilution 32.4 (30.3-35) mEq/L, p<0.004  *p=0.02; **p=0.001  B Atot variation from baseline throughout hemodilution (T0-T2-T4),  Baseline 14.1 (13.2-14.6), 8% dilution 12.9 (12.2-13.7), 13% dilution 11.9 (10.9-12.5) mmol/L, p<0.001  **p=0.001; *** p<0.001; ****p<0.001.  C pCO_2_ variation from baseline throughout hemodilution (T0-T2-T4),  Baseline 33.8 (31-37), 8% dilution 31 (29-36), 13% dilution 34.7 (31.7-38.1) mmHg, p=0.13 |
|   C  A |   B |
|  | **Figure S15 Effect of hemodilution on SIDa, pCO_2_ and Atot in NS population**  A SIDa variation from baseline throughout hemodilution (T0-T2-T4),  Baseline 33.7 (32-36), 8% dilution 33.4 (30-34), 13% dilution 31 (27-33.5) mEq/L, p<0.001  *** p<0.001; **p=0.006.  B Atot variation from baseline throughout hemodilution (T0-T2-T4),  Baseline 14.2 (13.9-15), 8% dilution 13 (12.4-13.7), 13% dilution 12 (11.5-12.5) mmol/L, p<0.001  **p=0.006; ****p<0.001.  C pCO_2_ variation from baseline throughout hemodilution (T0-T2-T4),  Baseline 33.4 (31-37), 8% dilution 32 (31-37), 13% dilution 35.4 (32-39) mmHg, p=0.14 |

**Figure S16 Albumin and Hemoglobin variation over time**

A

B

# **References**

1. The Acute Respiratory Distress Syndrome Network (2000) Ventilation with Lower Tidal Volumes as Compared with Traditional Tidal Volumes for Acute Lung Injury and the Acute Respiratory Distress Syndrome. New England Journal of Medicine 342:1301–1308. https://doi.org/10.1056/NEJM200005043421801

2. Stewart PA (1978) Independent and dependent variables of acid-base control. Respir Physiol 33:9–26. https://doi.org/10.1016/0034-5687(78)90079-8

3. Stewart PA (1983) Modern quantitative acid–base chemistry. Can J Physiol Pharmacol 61:1444–1461. https://doi.org/10.1139/y83-207

4. Constable PD (2001) Total weak acid concentration and effective dissociation constant of nonvolatile buffers in human plasma. J Appl Physiol 91:1364–1371. https://doi.org/10.1152/jappl.2001.91.3.1364

5. Siggaard-Andersen O (1971) An acid-base chart for arterial blood with normal and pathophysiological reference areas. Scand J Clin Lab Invest 27:239–245. https://doi.org/10.3109/00365517109080214

6. Gattinoni L, Carlesso E, Cadringher P, Caironi P (2006) Strong ion difference in urine: new perspectives in acid-base assessmentStrong ion difference in urine: new perspectives in acid-base assessment. Crit Care 10:137. https://doi.org/10.1186/cc4890

7. Inker LA, Eneanya ND, Coresh J, et al (2021) New Creatinine- and Cystatin C–Based Equations to Estimate GFR without Race. New England Journal of Medicine 385:1737–1749. https://doi.org/10.1056/NEJMoa2102953

8. Rein JL, Coca SG (2019) “I don’t get no respect”: the role of chloride in acute kidney injury. American Journal of Physiology-Renal Physiology 316:F587–F605. https://doi.org/10.1152/ajprenal.00130.2018
